# INNOVATE Research: Impact of a workshop to develop researcher capacity to engage youth in research

**DOI:** 10.1111/hex.13123

**Published:** 2020-09-09

**Authors:** Lisa D. Hawke, Karleigh Darnay, Marion Brown, Srividya Iyer, Shelly Ben‐David, Mohammad Khaleghi‐Moghaddam, Jacqueline Relihan, Skye Barbic, Lisa Lachance, Steve Mathias, Tanya Halsall, Sean A. Kidd, Sophie Soklaridis, Joanna Henderson

**Affiliations:** ^1^ Centre for Addiction and Mental Health Toronto Ontario Canada; ^2^ University of Toronto Toronto Ontario Canada; ^3^ Dalhousie University Halifax Nova Scotia Canada; ^4^ McGill University Montreal Quebec Canada; ^5^ ACCESS Open Minds Canada; ^6^ Douglas Hospital Research Centre Montreal Quebec Canada; ^7^ University of British Columbia Vancouver British Columbia Canada; ^8^ Foundry British Columbia Canada; ^9^ Wisdom2Action Halifax Nova Scotia Canada; ^10^ University of Ottawa Ottawa Ontario Canada

**Keywords:** capacity development, mental health, patient engagement, patient‐oriented research, youth, youth‐adult partnerships, youth engagement

## Abstract

**Background:**

Engaging youth in research provides substantial benefits to research about youth‐related needs, concerns and interventions. However, researchers require training and capacity development to work in this manner.

**Methods:**

A capacity‐building intervention, INNOVATE Research, was co‐designed with youth and adult researchers and delivered to researchers in three major academic research institutions across Canada. Fifty‐seven attendees participated in this research project evaluating youth engagement practices, attitudes, perceived barriers, and perceived capacity development needs before attending the intervention and six months later.

**Results:**

The intervention attracted researchers across various career levels, roles and disciplines. Participants were highly satisfied with the workshop activities. Follow‐up assessments revealed significant increases in self‐efficacy six months after the workshop (*P* = .035). Among possible barriers to youth engagement, four barriers significantly declined at follow‐up. The barriers that decreased were largely related to practical knowledge about how to engage youth in research. Significantly more participants had integrated youth engagement into their teaching activities six months after the workshop compared to those who were doing so before the workshop (*P* = .007). A large proportion (71.9%) of participants expressed the need for a strengthened network of youth‐engaged researchers; other future capacity‐building approaches were also endorsed.

**Conclusions:**

The INNOVATE Research project provided improvements in youth engagement attitudes and practices among researchers, while lifting barriers. Future capacity‐building work should continue to enhance the capacity of researchers to engage youth in research. Researchers notably pointed to the need to establish a network of youth‐engaged researchers to provide ongoing, sustainable gains in youth engagement.

## 1 BACKGROUND

Engaging individuals with lived experience in research projects related to their experiences and needs increases the quality of the research and the relevance to the target population.^1^, ^2^, ^3^, ^4^ This is reflected in a movement towards patient‐oriented research, supported by research bodies and policies around the world, including the National Institute for Health Research INVOLVE framework^5^ in the United Kingdom, the Canadian Strategy for Patient Oriented Research (SPOR),^6^ and Patient‐Centered Outcomes Research Network^7^ in the United States.

Indeed, engaging vulnerable community members in research is an ethical imperative. Literature has discussed the ethical considerations in working with vulnerable populations^8^, ^9^ in the context of historical inequities and the importance of building trusting relationships in research to avoid research‐related harms; engaging the target population supports the researchers in conducting research ethically and respectfully in a way that recognizes the population's needs, vulnerabilities and strengths.^10^, ^11^

Youth engagement in research is particularly important, given changing realities this emerging generation is facing and possible disconnection between young people and researchers. While youth have traditionally been research participants, youth engagement practices call for youth to be engaged in the research process as full partners.^3^ Youth engagement is valuable in any area of research regarding youth, ranging from mental health and substance use,^12^, ^13^, ^14^ health promotion^15^ and social inequity,^16^ to organizational change^17^ and educational reform.^18^ By engaging young people in all stages of a research project, from design and development through to knowledge translation, researchers can help ensure that the research they are conducting is relevant to the realities facing young people today.^3^, ^19^ Involving youth in research has benefits for the individual youth engaged, including knowledge acquisition and broad‐based development including leadership skills.^12^, ^20^ Youth engagement also has positive impacts on researchers as they gain opportunities for reciprocal learning.^12^, ^20^

Despite the benefits, many researchers working on youth‐relevant issues do not necessarily know how to engage youth thoroughly in their research activities, and capacity development efforts are therefore required. Unfortunately, there is a dearth of training available specifically targeting youth engagement education for Canadian researchers in academic settings. Researchers may look to the literature on patient engagement in research across the disciplines.^21^, ^22^, ^23^ They may also look to literature on the importance and value of youth engagement in research.^2^, ^24^ Literature describing examples of projects in which youth were engaged^14^, ^17^ and some general guidelines^25^ are also available. More systematic training opportunities are required, demonstrating how to engage youth in developmentally appropriate ways in complex research projects.

With a lack of training and experience, researchers beginning to engage youth may inadvertently tokenize or fail to engage them in authentic, meaningful manners, or omit youth engagement from some or all of the stages of their projects. In doing so, they may lose out on the considerable contributions that youth can make to research, while discouraging youth from becoming future ambassadors of research.

To increase the capacity of Canadian researchers to engage youth in their work, our team developed a training programme in youth engagement designed for researchers. The project is known as INNOVATE Research. It was developed by a pan‐Canadian team of researchers, youth, and engagement specialists from hospitals, universities and community organizations. Barriers and capacity development needs expressed prior to the workshop are presented in a companion manuscript.^26^ That paper shows that while interest in and attitudes towards youth‐engaged research were strong, skill development and practical training opportunities were found to be an important capacity development need.

### 1.1 Objective

This paper describes and examines the impact of the INNOVATE Research training on attendees’ youth engagement practices and attitudes, as well as their expressed barriers and further needs for capacity development. The training is expected to reduce perceived barriers and increase the application of youth engagement in research among participants.

## 2 METHOD

### 2.1 Participants

The sample consists of N = 57 researchers who attended one of three INNOVATE Research workshops and completed questionnaires before the workshop and six months later. Out of 84 participants who completed the pre‐workshop survey, 71 attended the workshops and were invited to complete the follow‐up survey, of whom 57 completed (82.3%).

### 2.2 Procedure

Recruitment for the workshop and the study was conducted via passive snowball sampling, by circulating a workshop flyer in the team's networks and to academics to solicit participation from researchers interested in youth engagement. Interested individuals contacted the research team for more information. Potential participants received an email informing them about the study and workshop. They were provided a survey link to the online survey in the weeks or days prior to the workshop, beginning with informed consent. They were informed that completing the pre‐workshop questionnaires entitled them to a $20 (50%) discount on the workshop and that study participants who attended the workshop would be invited to complete follow‐up questionnaires 6 months later for an honorarium equivalent to the remaining 50% of the workshop fee ($20). At the end of the workshop, attendees also completed a workshop feedback form. Research Ethics Board approval was obtained from academic institutions in the three sites of the workshop, specifically the Centre for Addiction and Mental Health, University of Toronto and the University of British Columbia, with approval as a quality improvement project from Dalhousie University.

### 2.3 Intervention

The INNOVATE Research project was designed by a pan‐Canadian team of researchers and youth co‐researchers representing academic research institutions and community organizations (Centre for Addiction and Mental Health, University of Toronto, McGill University, Dalhousie University, the University of British Columbia, the University of Ottawa, Wisdom2Action, Foundry, Frayme, ACCESS Open Minds, and the Douglas Hospital Research Centre). The core component of the intervention was a low‐barrier one‐day training activity, designed to be accessible to researchers with a range of experience in youth engagement, ranging from those just beginning to consider this type of work to those with concrete experience. The aim was to provide well‐rounded information on youth engagement, covering the importance and impact of youth engagement, how to prepare and plan for youth engagement in a research team, how to recruit youth to join a team and work with them as research partners, and how to evaluate and present the youth engagement activities. The workshops were co‐facilitated by youth and adult researchers. Delivery included presentations, concrete examples of youth‐engaged research projects, break‐out session discussions and practical activities focusing on building engagement into attendees research projects. The intervention also included a thorough curriculum that was developed and delivered, including a 76‐page guidebook^27^ (freely available by contacting the research team) and supplementary readings in the form of peer‐reviewed manuscripts on the topic.^14^, ^20^, ^25^ Following the workshops, attendees were invited to join two mentorship/coaching sessions delivered via webinar at approximately two‐month intervals to enhance and sustain their learnings. A one‐hour condensed version of the workshop was also provided for those who were not able to attend, in the form of a national webinar; the impacts of the national webinar on attitudes and practice change are not available.

### 2.4 Measures

The measurement set was developed by the research team with pan‐Canadian feedback through our professional networks. It was administered prior to the workshop and six months later, and included demographic and professional characteristics, questions regarding youth engagement practices, and a range of barriers and capacity development needs. The Service Provider Adopter and Innovation Characteristics Questionnaire (SPAICQ)^28^, ^29^ was also administered before the workshop and six months later. The SPAICQ is a 21‐item measure used in implementation studies to measure the degree of implementation of an innovation. With standardized stems, items were adapted to query about the implementation of youth engagement in research. Subscales include concern for youth engagement, self‐efficacy regarding youth engagement, the perceived complexity of engagement, the compatibility of engagement with one's way of working, and the relative advantage of engagement, calculated using average scores. All measures were administrated via the REDCap online data capture system.^30^ A pencil‐and‐paper post‐workshop evaluation form was also administered onsite immediately following the workshop to assess satisfaction.

### 2.5 Analyses

Descriptive statistics were compiled to describe the sample and their youth engagement practices at the pre‐workshop assessment time and six months after the workshop. McNemar's chi‐square tests were used to evaluate change over time on categorical variables. Repeated measures t tests were used for continuous variables. For workshop satisfaction, to examine whether the workshop was better suited to participants with different levels of education, independent sample Anovas were conducted for all variables across education levels of Bachelor's or less (N = 19), Master's, (N = 21) and Ph.D/MD (N = 21) (3 missing); comparisons of satisfaction by site were also conducted (Toronto N = 30, Halifax N = 18 and Vancouver, N = 17). Missing data were minimal. For SPAICQ scores, mean scores were employed to minimize the impact of missing data. For categorical data, percentage scores represent the percentage of the total number of respondents to that item. Alpha values of < 0.05 were interpreted as significant. SPSS 25 was used.^31^ Effect sizes were calculated using G*Power.^32^

## 3 RESULTS

Participant characteristics are described in Table 1. Participants represented a broad range of disciplines and career levels. The majority were female and relatively early in their careers.

**Table 1:** Participant characteristics (N = 57)

Characteristic		N (%)
Age	20‐29	23 (40.4%)
	30‐39	19 (33.3%)
	40‐49	10 (17.5%)
	50+	5 (8.8%)
Sex	Male	6 (10.5%)
	Female	51 (89.5%)
	Not male or female	0 (0.0%)
Primary position	University Professor/Administrator	8 (14.5%)
	Community/hospital‐based researcher	12 (21.8%)
	Trainee (PDF, PHD, Other trainee)	19 (34.5%)
	Research staff	10 (18.2%)
	Other	6 (10.9%)
Education	Bachelor's or less	13 (22.8%)
	Master's	24 (42.1%)
	PhD, MD	16 (28.1%)
	Other	4 (7.0%)
Primary discipline	Psychology	21 (36.8%)
	Social Work	10 (17.5%)
	Sociology	10 (17.5%)
	Medicine (psychiatry or other)	8 (14.0%)
	Other Health	12 (21.1%)
	Other Social Sciences	7 (12.3%)
	Other	6 (10.5%)
Years of experience in youth‐relevant issues	Less than one Year	9 (16.1%)
	1‐5 Years	28 (50.0%)
	6–10 Years	13 (23.2%)
	11+	6 (10.7%)
Percentage of time spent on research	0	3 (5.3%)
	1%‐25%	17 (29.8%)
	26%‐50%	11 (19.3%)
	51%+	26 (45.6%)

The research and engagement profiles of participants are presented in Table 2. Results show that researchers considered themselves significantly more familiar with youth engagement six months after the workshop. In a six‐month period, there was no change in the numbers engaging youth in practice or planning for youth engagement in grants. However, participants were significantly more likely to be including youth engagement in their teaching activities. There was also an increase in the number of participants reporting that they were engaging youth in the form of co‐presenting findings at conferences.

**Table 2:** Research and engagement profiles of participants before the workshop and six months later, with McNemar chi‐square significance value

Characteristic		Pre‐workshop	6 month follow‐up	*P*
		N (%)	N (%)	
Familiarity with youth‐engaged research	Very Familiar	19 (33.3%)	21 (37.5%)	.0499
	Somewhat familiar	30 (52.6%)	32 (57.1%)	
	Not very familiar	8 (14.0%)	3 (5.4%)	
Currently do stakeholder engaged research		38 (66.7%)	36 (63.2%)	.549
Currently do youth‐engaged research		33 (57.9%)	36 (63.2%)	.791
Number of projects on that include youth engagement	0	17 (30.9%)	19 (33.3%)	.644
	1	23 (41.8%)	23 (40.4%)	
	2+	15 (27.3%)	15 (26.3%)	
Number of grants planned	0	27 (49.1%)	32 (57.1%)	.093
	1	22 (40.0%)	16 (28.6%)	
	2+	6 (10.9%)	8 (14.3%)	
Teaching	Yes	3 (6.0%)	10 (18.9%)	.007
	Working on it	3 (6.0%)	10 (18.9%)	
	No, considering	6 (12.0%)	10 (18.9%)	
	No	38 (76.0%)	23 (43.4%)	
How youth are engaged	Initial planning (identify research question, writing grant)	14 (24.6%)	15 (26.3%)	1.000
	Design (methodology, recruitment strategies and measurements selection)	28 (49.1%)	30 (52.6%)	.824
	Co‐analysing/interpreting findings	21 (36.8%)	18 (31.6%)	.648
	Identification of target audiences and knowledge translation strategies	26 (45.6%)	31 (54.4%)	.359
	Co‐developing knowledge translation materials	17 (29.8%)	21 (36.8%)	.219
	Co‐presenting at conferences	14 (24.6%)	22 (38.6%)	.008
	Co‐authoring manuscripts	8 (14.0%)	14 (24.6%)	.146

Table 3 presents the attitudes of participants regarding youth engagement before and six months after the workshop. Results show a significant increase in their sense of self‐efficacy with regard to implementing youth engagement. There was no significant change in any of the other attitudes regarding youth engagement.

**Table 3:** SPACIQ mean scores (with standard deviation) before the workshop and 6 months later, with repeated measures t tests and effect sizes

Subscale	Pre‐workshop	6 month follow‐up	*t*(56)	*P*	*d*
	M (SD)	M (SD)			
Concern	4.5 (0.7)	4.6 (0.4)	0.373	.711	0.049
Self‐Efficacy	3.5 (0.6)	3.7 (0.7)	2.161	.035	0.286
Complexity	3.0 (0.5)	3.1 (0.7)	1.146	.256	0.152
Compatibility	4.1 (0.6)	3.9 (0.7)	1.977	.053	0.262
Relative Advantage	4.2 (0.8)	4.3 (0.6)	1.710	.093	0.227

Barriers to youth engagement experienced by participants are presented in Table 4. Significant decreases were found on four of 11 barriers six months after the workshop. The barriers that were most highly endorsed prior to the workshop significantly declined: fewer participants reported that they did not know how to engage youth, how to get a representative group of youth together, or how to prepare youth to engage research. Uncertainty about ethical considerations also declined. One barrier was reported significantly more frequently at the six month follow‐up assessment: not having the time or human resources to support youth engagement.

**Table 4:** Barriers identified by participants before the workshop and six months later, with McNemar chi‐square significance value

Barrier	Pre‐workshop	6 month follow‐up	*P*
	N (%)	N (%)	
Not sure how to engage youth on a practical level	26 (45.6%)	11 (19.3%)	<.001
Don't know how to get a representative group of youth together	24 (42.1%)	12 (21.1%)	.017
Don't know how to prepare youth to engage in research in this way	24 (42.1%)	13 (22.8%)	.027
Don't have funding to support this	21 (36.8%)	24 (42.1%)	.607
Unsure about the ethical considerations of engaging youth	20 (35.1%)	8 (14.0%)	.012
Don't have time or human resources to support this	9 (15.8%)	22 (38.6%)	.004
Department/university doesn't recognize the value	6 (10.5%)	10 (17.5%)	.219
Other institutional barrier	6 (10.5%)	9 (15.8%)	.508
Not sure I can appropriately relate to youth or communicate with them effectively	3 (5.3%)	9 (15.8%)	.109
Not relevant to the type of research I do	2 (3.5%)	4 (7.0%)	.687
Not interested in working in this way	0 (0.0%)	0 (0.0%)	n/a

The capacity development needs of participants before the workshop and six months later are presented in Table 5. One of the most frequently identified needs prior to the workshop, specifically, additional training, was significantly reduced six months later. The remaining capacity development needs did not change significantly. At follow‐up, the most highly endorsed capacity development needs were a strengthened network of youth‐engaged researchers, greater funder appreciation of youth engagement and greater institutional appreciation of youth engagement.

**Table 5:** Capacity development needs endorsed by participants before the workshop and six months later, with McNemar chi‐square significance value

Capacity development need	Pre‐workshop	6 month follow‐up	*P*
	N (%)	N (%)	
Strengthened network of youth‐engaged researchers	43 (75.4)	41 (71.9)	.815
Additional training	39 (68.4)	21 (36.8)	.002
Ongoing consultation/supervision	25 (43.9)	19 (33.3)	.238
Greater funder appreciation of youth engagement	25 (43.9)	31 (54.4)	.263
Greater institutional appreciation of youth engagement	22 (38.6)	24 (42.1)	.832
Enhanced curriculum	21 (36.8)	16 (28.1)	.359
Online training	20 (35.1)	19 (33.3)	1.000

### 3.1 WORKSHOP SATISFACTION

Workshop evaluation results are presented in Table 6. Workshop evaluations were available for 51 participants (89.5%). Results show that, on a 1‐5 scale, participants were highly satisfied with the presenters, the workshops’ achievement of the learning objectives and the workshops overall. There were no significant differences between satisfaction based on level of education (*p* range 0.19 to 0.86) or based on site (*p* range 0.12 to 0.91). Among attendees, 94.1% stated that the workshop would change their practice, while 96.1% stated that they intended to use the INNOVATE Research curriculum provided.

**Table 6:** Ratings of satisfaction with workshop presenters and activities

Satisfaction		M (SD)
Presenter ratings	Clarity of presenter	4.8 (0.4)
	Relevance to practice	4.7 (0.4)
	Stated objectives were met	4.8 (0.4)
	Opportunity for active learning	4.6 (0.6)
	Balanced and unbiased	4.8 (0.4)
Learning objectives	Can identify practical steps to engage youth	4.5 (0.7)
	Can describe the value of youth engagement	4.8 (0.5)
	Can discuss engagement in different methodologies	4.4 (0.7)
Overall evaluation	Satisfied with the workshop	4.8 (0.5)
	Learned something new	4.7 (0.7)
	Relevant and useful	4.7 (0.6)
	Provided an opportunity to network	4.5 (0.7)
	Sufficient time for participation, active learning	4.4 (0.7)
	Would recommend to others	4.8 (0.4)

#### 3.1.1 Use of additional intervention components

The two coaching sessions held after the workshop were attended by 15 participants and 4 participants, respectively. The live national webinar was attended by 24 people. The 76‐page curriculum was distributed to all workshop attendees in hard copy, as well as at supplementary knowledge translation events and presentations. Outside of the workshops, the curriculum was downloaded from the website by 131 people between May 2019 and July 2020.

## 4 DISCUSSION

This study evaluated the impact of an intervention for researchers to increase capacity to engage youth in research activities. Three workshops were delivered to researchers across three major Canadian cities with multiple academic research institutions. The workshop attracted researchers across career levels, roles and disciplines. Participants were highly satisfied with the workshop activities across multiple metrics. Six month follow‐up evaluations showed several significant changes: among attendees, more were integrating youth engagement into their teaching activities and more were engaging youth as co‐presenters at conferences, and their familiarity with youth‐engaged research had increased. Attitudes, which were very positive prior to the workshop, remained largely the same, although the sense of self‐efficacy improved. Multiple barriers decreased, and the expressed need for training declined, although the need for a strengthened network of youth‐engaged researchers remained very high. Despite strong satisfaction, a large majority of participants expressing interest in developing a network, and a third of participants expressing interest in additional training and ongoing consultation. Modest engagement with the coaching sessions was observed.

Viewing engagement as an ethical imperative,^10^, ^11^ it is essential that ongoing work continues to examine manners of increasing researcher capacity to engage youth authentically and collaboratively, building trusting relationships with youth who are meaningfully engaged. This process will provide gains for researchers and youth in a reciprocal manner, improving the quality, relevance and trustworthiness of the research and improving trust in researchers and research activities. The high levels of practical, knowledge‐based barriers reported prior to the intervention demonstrate that, even among researchers who are interested in youth engagement, practical skills are lacking. However, training opportunities designed to address practical knowledge gaps can provide incremental gains that will position researchers to embark on a path of youth‐engaged research, with all of the benefits that this way of working has for researchers and youth alike.

Studies evaluating more intensive training opportunities in academic and community or patient collaborations, with multiple components ranging from coursework to mentorship, have found positive changes over the course of the programming in terms of knowledge building, competence and self‐efficacy.^33^, ^34^ These studies have advocated for multi‐component training programmes, with opportunities to apply new skills. Unfortunately, intensive programmes can accommodate and are only of interest to smaller numbers of people over greater periods of time due to the availability constraints of researchers. Researchers may be advantaged by initial training that reduces perceived barriers, increases motivation and encourages participants to progress to a higher level of interest and, consequently, to apply themselves to a longer‐term, intensive process. The modest and declining participation in the follow‐up coaching sessions observed in this project point to the importance of strong initial, flexible training components, as the engagement of trainees may vary over time.

Building skills, self‐efficacy and sustainability through mentorship, while reducing barriers are important building blocks of capacity in research.^35^, ^36^ In the INNOVATE Research project, the sense of self‐efficacy increased after the intervention; this increase may be associated with the decline in multiple barriers, including the three most highly endorsed barriers regarding the practical application of youth engagement. Importantly, one barrier increased: significantly more participants endorsed that they do not have the time or human resources to support youth engagement. While the workshop activities aimed to increase capacity and reduce barriers, the higher endorsement of this barrier after the workshop suggests that meaningful learnings were achieved. Engaging youth effectively in research requires an investment of time and human resources,^37^, ^38^ sometimes slowing the progress towards key project deliverables, while improving the quality. It is important that researchers be aware of the time and human resource investment required prior to beginning the youth engagement process in order to ensure adequate support for youth and adjust project timelines accordingly. Greater institutional support for youth engagement, including recognition of the time commitments, together with greater networking activities, may help reduce this barrier.

Future capacity‐building initiatives aiming to increase youth engagement in research should consider increasing networking and mentoring opportunities, improving infrastructure, enhancing sustainability efforts and providing flexible ongoing professional development opportunities.^35^, ^36^ When aiming to increase capacity, initial gains may be small, as substantial gains in measurable research outputs and the development of a local research engagement infrastructure take time to emerge. However, small initial gains may lay the foundation for larger gains with time and experience.^35^ For example, sharing knowledge with junior researchers is considered an important capacity‐building step.^35^ That aim was achieved in the current study in two ways: many workshop attendees were relatively junior in their careers, and after the workshop, more participants reported integrating youth engagement into their teaching. Trainee capacity development may provide benefits over the longer term, as trainees who begin their research careers with an understanding of youth engagement may be more likely to practice their work in this manner. While researcher capacity development can occur as part of trainees’ formal training, substantial gains can also be made through ongoing professional development opportunities for established professionals.^36^, ^39^ Policy factors can also influence the success of capacity‐building initiatives,^35^ reflected here in participants’ expressed need for greater institutional and funder support, which may help make practice gains more feasible and sustainable. Indeed, culture changes can be required to increase research capacity.^36^ The literature on building capacity for health research in general suggests that traditional metrics such as grant applications and peer‐reviewed publications do not capture the scope of actual advancement in research capacity; success at building capacity should focus on effects on the initiatives, services or activities that arise from the capacity building and the recipients or participants in those endeavors.^35^

Among capacity development needs, the need for a strengthened network of youth‐engaged researchers was very highly endorsed. The Canadian Institutes of Health Research, in partnership with the Strategy for Patient‐Oriented Research, recently released a call for proposals to develop a National Training Entity in Patient Engagement.^40^ This upcoming Entity will bring together researchers engaging individuals with lived experience across the health sectors, together with the individuals themselves, to build research capacity through a variety of means. That future Entity holds the potential to provide substantial gains in patient engagement, including youth engagement, by scaling capacity development initiatives such as INNOVATE Research, continuing to reduce barriers and prioritizing the areas of capacity development highlighted in the current project.

## 5 LIMITATIONS

This study was conducted in three urban centres in Canada associated with major academic research institutions. Results may not be generalizable to other jurisdictions and smaller academic research institutions; further scaling and evaluation initiatives are therefore required. In addition, this study did not evaluate the impact of the training on the youth ultimately engaged in the research conducted by the intervention participants. The participants were researchers with an interest in youth engagement, and many had experience in youth engagement; effects might be different among researchers without this interest and experience, or in fields in which youth engagement is less prevalent. The majority of participants were also female. Larger, more diverse samples are required in future research and evaluation activities to understand differential impacts for population subgroups. Future work should also focus on engagement training for the youth the teams engage. Despite these limitations, this study had some unique strengths. The intervention was co‐developed, co‐delivered and documented in the form of an implementation workbook,^27^ as well as evaluated by a pan‐Canadian team of academics and youth. The workshop content integrated evidence from across diverse domains with concrete examples from major patient‐oriented research initiatives in Canada, leveraging previous research investments in patient‐oriented research. Future research should extend the evaluation of initiatives aiming to develop capacity in youth‐engaged research by evaluating the impacts on the subsequent research activities by participants, the youth engaged, and on the outcomes of the research activities. Future research activities should also evaluate the longer‐term impact of youth engagement capacity‐building activities on changes in the application of youth engagement in research, as measureable changes in research deliverables take time to emerge.

## 6 CONCLUSIONS

Given the substantial benefits associated with engaging youth in research about youth‐related health needs, concerns and interventions, it is important to increase the capacity of researchers to work in this manner. The INNOVATE Research project is a highly appreciated youth‐engaged capacity‐building initiative that provides gains in this area by building knowledge and reducing barriers. Future work and scaling of capacity development initiatives are needed to continue to enhance capacity, to establish a network of youth‐engaged researchers and to provide ongoing, sustainable gains in youth engagement among researchers.

## CONFLICT OF INTEREST

None.

## AUTHOR CONTRIBUTIONS

LH supported the design and execution of the study, analysed the data, interpreted the results and drafted the manuscript. KD, MB, SI, MKM, JR, SB, LL, SM, TH, SK and SS supported the design and execution of the study, co‐interpreted the results and reviewed the manuscript. JH led the project, supported the design and execution of the study, co‐interpreted the results and reviewed the manuscript.

## Data Availability

The data that support the findings of this study are available from the corresponding author upon reasonable request.
